# Safety and Efficacy of Influenza Vaccination in Kidney Graft Recipients in Late Period After Kidney Transplantation

**DOI:** 10.3390/vaccines13020189

**Published:** 2025-02-14

**Authors:** Anna Zawiasa-Bryszewska, Maja Nowicka, Monika Górska, Piotr Edyko, Krzysztof Edyko, Damian Tworek, Adam Antczak, Jacek Burzyński, Ilona Kurnatowska

**Affiliations:** 1 Department of Internal Medicine and Transplant Nephrology, Medical University of Lodz, 90-419 Lodz, Polandpiotredyko@gmail.com (P.E.);; 2 Student Scientific Society Affiliated with the Department of Internal Medicine and Transplant Nephrology, Chair of Pulmonology, Rheumatology and Clinical Immunology, Medical University of Lodz, 90-419 Lodz, Poland; 3 Department of General and Oncological Pulmonology, Medical University of Lodz, 90-419 Lodz, Poland; 4 Department of Statistics and Translational Medicine, Medical University of Lodz, 90-419 Lodz, Poland

**Keywords:** influenza virus, transplant recipients, antibody response

## Abstract

Background/Objectives: Influenza is a viral infection affecting up to 20% of the general population annually. Solid organ transplant recipients have a higher morbidity and mortality risk, as well as a greater likelihood of severe disease complications. Vaccination against the influenza virus is a safe and recommended prophylaxis; however, immunosuppression and high comorbidity burdens impair the immune response. We assessed the efficacy, safety, and humoral response to influenza vaccine in a population of kidney transplant recipients (KTx). Methods: Adult KTx recipients at least 6 months post-KTx were divided into vaccinated (vKTx) and non-vaccinated (nvKTx) groups based on consent for vaccination. The vKTx group received one dose of quadrivalent split virion inactivated vaccine (Vaxigrip Tetra Sanofi Pasteur). Subjective symptoms and side effects were recorded in paper journals. Antibody levels were assessed with ELISA prior to and 3 months following vaccination. Serum creatinine and proteinuria were assessed prior to vaccination as well as 3 and 6 months after. Results: Of 450 recruited KTx recipients, 91 in the vKTx group and 36 in the nvKTx group of comparable age, KTx vintage, and graft function were included in the study. Graft function and proteinuria remained stable in both groups. The vKTx group experienced no severe adverse events. The most common complaints were general malaise (20.5%) and injection site pain (10.3%). Overall infection rates were comparable, yet the vKTx group experienced significantly fewer serious infections (11.4% vs. 32.3%, *p* = 0.01); the vKTx group showed a greater increase of Influenza A IgM (*p* = 0.05) and Influenza B IgG (*p* = 0.01) compared with the nvKTx group. Conclusions: Influenza vaccination prevents severe infections in KTx recipients, with good serological response and no impact on graft function or severe adverse events.

## 1. Introduction

Influenza remains a significant and persistent health concern. Each year, seasonal influenza virus affects up to 20% of the population, causing highly contagious symptoms which predominantly include cough, sore throat, and rhinitis accompanied by fever, headache, general malaise, and diffuse myalgia [1]. Influenza accounts for relatively few deaths as the underlying cause, but frequent complications may have profound clinical impact [2].

Solid organ transplant (SOT) recipients, usually affected by impaired immune systems resulting from immunosuppressive treatment, frequently suboptimal graft function, and concomitant diseases, are at high risk of significant morbidity and mortality following influenza infection [3,4]. Complications arising from influenza infection include secondary bacterial pneumonia as well as extrapulmonary manifestations such as septic shock, myocarditis, cardiomyopathy, myositis, seizures (both febrile and non-febrile), encephalopathy, graft rejection, or even death [5,6]. Thus, protection against the influenza virus is crucial in this population.

The influenza virus is a negative-sense, single-stranded, and segmented RNA orthomyxovirus that circulates primarily in the autumn and winter months, with A/H1N1, A/H3N2, and B being the predominant seasonal strains [7].

Mass vaccination is the most effective prophylaxis for protecting a population during an influenza virus pandemic [8,9]. Protection as a result of influenza vaccination is achieved through both humoral and cellular immune cruciality, typically evaluated by measuring vaccine-specific antibody levels. In healthy adults, it takes approximately 14 days post-vaccination to reach the peak antibody protection [9].

Many infections in SOT recipients may be prevented by proper prophylaxis and vaccines, which should be widely recommended. Inactivated vaccines are safe to administer starting 3 months post-transplant, except for influenza vaccines, which can be given as early as one month after transplantation [10]. Influenza is the most prevalent vaccine-preventable infection in SOT recipients in the first 5 years post-transplant [7]. Unfortunately, up to 50% of SOT patients refuse vaccination, fearing mainly that excessive stimulation of the immune system might be associated with an increased risk of graft rejection [11,12].

Many studies have shown that the response to vaccines in SOT may be decreased and that it is associated with the timing of vaccination relative to transplantation, the type of immunosuppressive regimen, and graft function [7,8]. Most studies evaluating the effects of seasonal influenza vaccination do not find the risk of graft rejection or dysfunction increased [13], although they are mainly just descriptive, evaluating both the immunogenicity and safety profile of the vaccines only in small case series [8].

The aim of our study was to evaluate the clinical effectiveness, safety, tolerability as well as immunogenicity and the antibody response to influenza vaccination in recipients in late period after kidney transplantation (KTx).

## 2. Materials and Methods

A prospective open-label study was conducted at two post-transplant outpatient clinics in Central Poland between October 2018 and May 2019. The study was approved by the Bioethical Committee (Approval No. RNN/338/18/KE from 16 October 2018).

### 2.1. Patients

Included in the study were adult recipients at least 6 months post-KTx, with stable graft function (eGFR MDRD >15 mL/min/1.73 m^2^ and 3-month variations of serum creatinine level < 0.3 mg/dL and eGFR < 20%), no contraindications for vaccination, and who provided written informed consent.

Exclusion criteria were known hypersensitivities to any component of the vaccine formulation, history of acute graft rejection in the last 6 months, history of non-compliance, and unwillingness or inability to follow all study procedures. Patients presenting with any infection symptoms at the time of planned vaccination had their vaccination delayed until full clinical recovery.

### 2.2. Study Design

Eligible patients were divided into a study group (vaccinated KTx recipients, vKTx), based on their willingness to be vaccinated, and a control group (non-vaccinated KTx recipients, nvKTx). The vKTx group received one intramuscular injection of quadrivalent Vaxigrip Tetra Sanofi Pasteur (split virion, inactivated) in the deltoid muscle of their non-dominant arm in October and November 2018.

### 2.3. Clinical Data

Data regarding patients’ sex, age, anthropometric data (weight, height), medical history including comorbidities (diabetes, ischemic heart disease, lung disease, stroke, myocardial infarction), transplantation history, immunosuppression regimen, and previous influenza vaccination history, were collected from the outpatient clinic medical records.

### 2.4. Self-Observational Diaries

All study procedures, including vaccinations, were performed during routine check-ups in posttransplant outpatient clinics.

On the day of study commencement (time point 1; TP1), which was also the day of vaccination (vKTx) and/or consent to participate in the observational group without vaccination (nvKTx), all patients were given self-observational diaries. Participants were instructed to complete the diaries which recorded the monitoring of potential adverse effects of the vaccination (vKTx) and weekly occurrences of flu-like symptoms during the period of 24 weeks (vKTx and nvKTx).

The adverse effects of vaccination were classified as either local reactions (pain, redness, and swelling at the injection site) or systemic reactions (general malaise, weakness, fever—defined as a body temperature above 38 °C, headache, muscle pains, nausea, vomiting, and anaphylaxis).

The monitoring involved recording flu-like symptoms such as general malaise, weakness, fever, chills, headache, sore throat, coughing, nasal discharge, muscle pain, and anorexia, as well as the occurrence of similar symptoms in patients’ household members, and also their treatment, particularly regarding the antibiotic use and the need for hospitalization. Infections were defined as ‘mild’ with the occurrence of two or more flu-like symptoms, with at least one lasting over 3 days, and as ‘severe’ with the need for hospitalization and/or fever with at least one flu-like symptom lasting over 3 days.

### 2.5. Laboratory Data

Kidney graft function assessed using serum creatinine (eGFR), proteinuria, and full blood count were assessed prior to vaccination (TP1), 3 months after (time point 2: TP2), and 6 months after (time point 3: TP3). Kidney graft function (glomerular filtration rate) was calculated from serum creatinine (sCr) using the MDRD formula [14]. Proteinuria was considered clinically significant at >0.5 G/L in spot morning urine. Leucopenia was defined as white blood cell count < 4.0 × 10^9^ /L, neutropenia as neutrophil count < 1.8 × 10^9^ /L, and thrombocytopenia as platelet count < 150,000 G/L.

### 2.6. Serological Data

Patients provided additional informed consent for the analysis of serum antibodies.

Serum samples were collected before (TP1) and 3 months after (TP2) vaccination and stored at minus 70 °C for further influenza virus antibody measurement. The immune response was assessed using the IBL International brand Anti-Influenza A virus and Anti-Influenza B virus IgM and IgG antibodies enzyme immunoassays (ELISA) kits with 95% sensitivity and 95% specificity. Antibody levels below 8 U/mL were considered negative, levels above 12 U/mL were considered positive, while levels of >8 U/mL and <12 U/mL were interpreted as inconclusive.

### 2.7. Statistical Analysis

Continuous variables with a non-normal distribution were presented using the median with the 25% and 75% quartiles (Q1–Q3). Nominal variables were presented using counts and percentages. Nominal variables were compared using Chi-squared tests with appropriate corrections. Continuous variables in independent pairs were compared using the non-parametric Mann–Whitney U test. Statistically significant results were presented using box-and-whisker plots. Continuous variables in repeated measurements were compared using the non-parametric Wilcoxon test for paired samples. Results are presented using line plots of case profiles.

To assess the effectiveness of vaccination, a multivariate logistic regression model with stepwise backward variable selection was used. Results are presented using odds ratios (OR) with 95% confidence intervals (95% CI). The quality of the model was assessed using the ROC curve with the area under the curve (AUC) with 95% CI, as well as sensitivity and specificity. Sensitivity and specificity were determined based on the Youden index. To assess the impact of vaccination and clinical factors on the change in antibody levels between the initial and final points, a repeated measure ANOVA test was used. The given *p*-value refers to the interaction of time and the given factor.

A significance level of *p* = 0.05 was adopted. The analysis was conducted using STATISTICA software (StatSoft, TIBCO, Cracow, Poland) version 13.3 and RStudio.

## 3. Results

**Figure 1:**
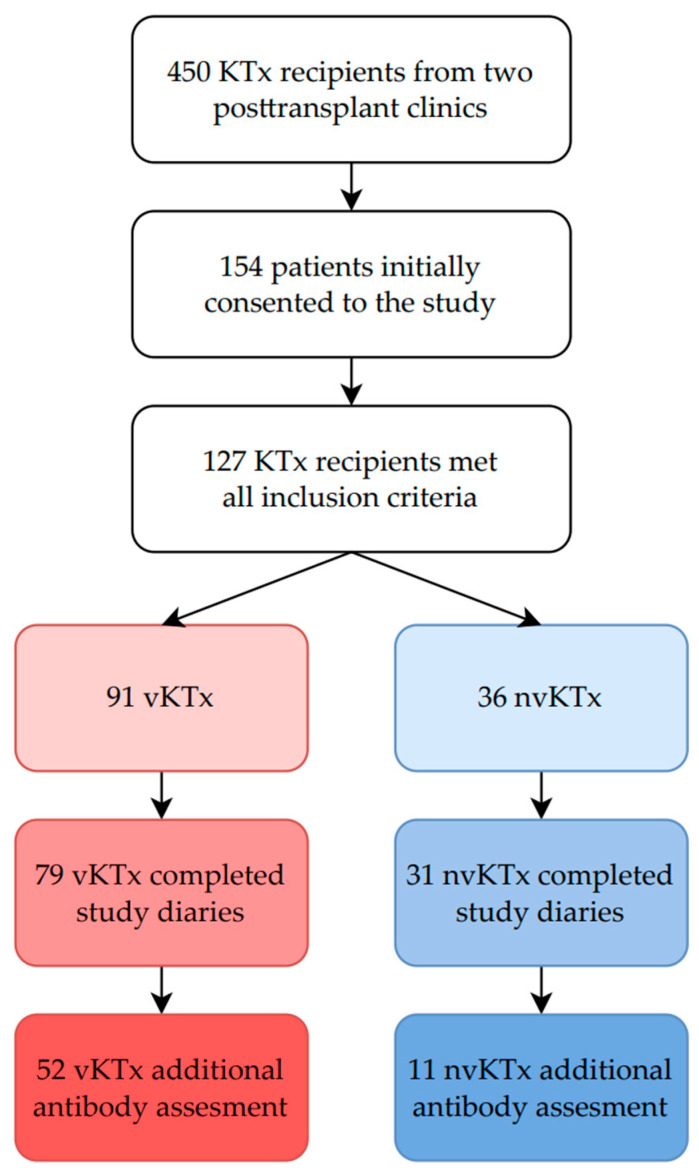
Study recruitment flowchart.

Out of 450 KTx recipients under the care of our post-transplant outpatient clinics, 154 initially consented to all study procedures; 127 patients met all inclusion criteria, resulting in 91 vKTx patients and 36 nvKTx patients being included in the study. The final analysis included 110 KTx patients who completed self-observational diaries, including 79 vKTx patients and 31 nvKTx patients (Figure 1).

### 3.1. Baseline Characteristics

The vKTx group (mean age 55.3 (44.8–62.5) years; mean KTx vintage 5.8 (3.1–8.0) years), and the nvKTx group (mean age 53.6 (44.9–60.7) years; mean KTx vintage 6.8 (3.9–11.0) years) did not differ significantly in regard to age, time after transplantation, kidney graft function (eGFR) or history of previous influenza vaccination. The vKTx group consisted of significantly more males (*p* = 0.02) and presented with more frequent clinically significant proteinuria at baseline (*p* = 0.014). The majority (*n* = 91, 82.7%) of the study population, both vKTx and nvKTx groups, received standard triple immunosuppressive therapy, with steroids at a maintenance daily dose of 5 mg prednisone, mycophenolate mofetil (MMF)/mycophenolate sodium (MPS), and a calcineurin inhibitor (cyclosporine A (CsA), tacrolimus (TAC)) or mTOR inhibitor—everolimus; 17 patients (15.5%) received double immunosuppressive therapy without MMF/MPS and 2 patients (1.8%) received double immunosuppressive therapy without steroids; no patients on monotherapy were included to the study. All included patients had a C0 level of calcineurin inhibitor or mTOR inhibitor adequate to recommendations for the time after KTx (for CsA 75–125 ng/mL, TAC 5–8 ng/mL, everolimus 3–8 ng/mL). A total of 36% (*n* = 32) of patients included in the study reported influenza vaccination in the previous year in relation to the study period. Complete study population characteristics are presented in Table 1 and Table 2.

### 3.2. Safety

The most frequent adverse effects of vaccination included general malaise (20.5%, *n =* 16), injection site pain (10.3%, *n =* 8), myalgia (7.7%, *n =* 6), injection site redness (5.1%, *n =* 4) and headache (5.1%, *n =* 4). There were no observed severe anaphylaxis reactions, and only one vaccinated patient reported a fever episode.

We did not observe any significant difference between the vKTx and nvKTx groups in changes (TP1/TP2 and TP1/TP3) of eGFR: -1.9 (−5.2–1.2) vs. −0.7 (−0.3–1.8) mL/min/1.73 m^2^, *p* = 0.30 and −1.0 (−5.0–2.0) vs. 1.0 (−3.0–5.0) mL/min/1.73 m^2^; *p* = 0.07, respectively, of sCr: 0.06 (−0.04–0.16) vs. 0.01 (−0.04–0.08) mg/dL, *p* = 0.16 and −1.27 (−1.71–−0.90) vs. −1.07 (−1.40–−0.89) mg/dL, *p* = 0.16, respectively, nor in the occurrence of clinically significant proteinuria: 0% vs. 7.1%, *p* = 0.60 and 7.4% vs. 5.8%, *p* > 0.99, respectively. None of the vKTx group experienced leucopenia, neutropenia, or thrombocytopenia after vaccination.

### 3.3. Efficacy

The vKTx group was less likely to develop severe infections compared with the nvKTx group (11.4% vs. 32.3%, *p* = 0.01, OR = 0.27, 95% CI 0.10–0.75; 0.15 vs. 0.35 of severe infections per patient, *p* = 0.01). However, there was no statistical difference in the rates of mild infection, infection development as the total (mild or severe), and hospitalization due to infectious reasons. Notably, the simultaneous occurrence of similar infectious symptoms among patients’ family and/or coworkers was reported significantly less frequently by the vKTx group (24.4% vs. 57.1%, *p* = 0.013). Moreover, the vKTx group tended to use antibiotics less frequently than the unvaccinated group, though this difference did not reach the point of statistical significance (Table 3).

To identify factors associated with severe infection occurrence, we developed a multivariable logistic regression model with stepwise variable selection. In our model, vaccination was associated with decreased odds of severe infection development, whereas the history of diabetes and lung disease, as well as a longer time after transplantation, were associated with higher odds of severe infection. The model demonstrated a sensitivity of 88.9%, specificity of 66.7%, and ROC AUC 0.81, 95% CI (0.70–0.92) for predicting severe infections (Table 4).

**Figure 2:**
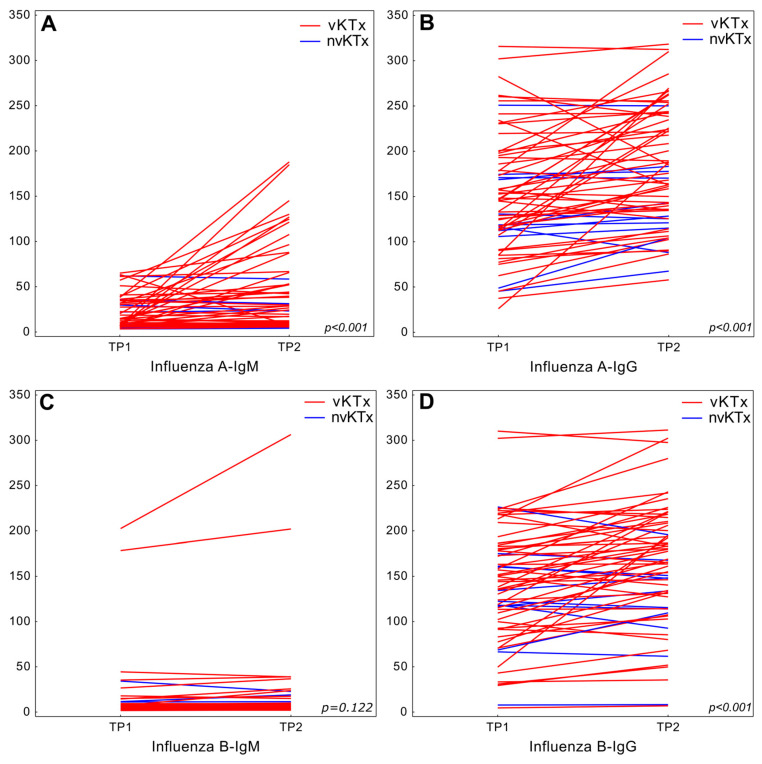
Change of Anti-Influenza virus antibody levels between TP1 and TP2 visits in the entire study population (vKTx+ nvKTx). Anti-Influenza A virus antibodies in (**A**) and IgG (**B**) class. Anti-Influenza B virus antibodies in IgM (**C**) and IgG (**D**) class.

Additional consent for the analysis of antibodies was given by 63 KTx patients (52 vKTx patients, 11 nvKTx patients). In the whole population, between TP1 and TP2 we observed a statistically significant increase in both IgM and IgG antibodies against Influenza A virus (*p* < 0.001). For Anti-Influenza B virus, only IgG antibodies showed a significant increase (*p* < 0.001) (Figure 2).

**Figure 3:**
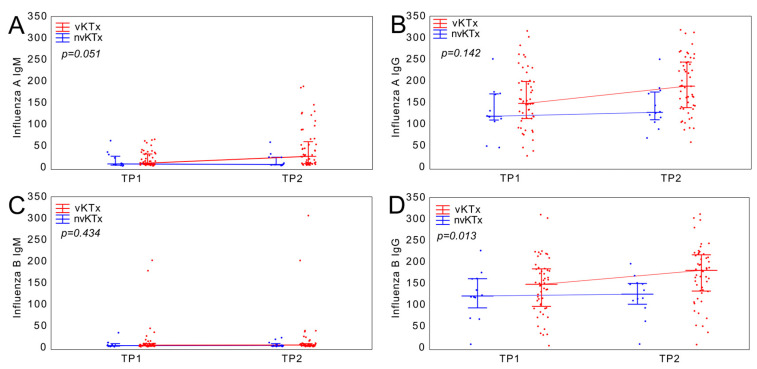
Change of Anti-Influenza virus antibody levels in regard to vaccination status.

The elevation of antibody level was significantly greater in the vKTx group than in the nvKTx group for the Anti-Influenza A virus (*p* = 0.05) and the Anti-Influenza B virus IgG (*p* = 0.01) (Figure 3).

Anti-Influenza A antibodies in IgM (A) and IgG (B) class. The line denotes the median, whereas whiskers span the interquartile range (Q1–Q3). Anti-Influenza B antibodies in IgM (C) and IgG (D) class. The line denotes the median, whereas whiskers span the interquartile range (Q1–Q3).

Almost all vKTx and nvKTx patients presented positive Anti-Influenza virus A and B IgG antibody titers at TP1. At TP2, we observed a slight increase in patients with positive IgM Anti-Influenza A virus antibody titer in the vKTx group (Table 5). Only 1 vKTx patient remained IgG negative throughout the observation period—a 26-year-old male, 9.5 years following KTx, treated with prednisone, cyclosporine, and mycophenolate mofetil (2 × 1000); comorbidities included hypertension; he had no prior history of influenza vaccination and has experienced two minor infections in the observation period.

No statistically significant differences were observed in antibody level changes (IgM and IgG, Anti-Influenza A virus, and Anti-Influenza B virus) with regard to patients’ sex or comorbidities. There were also no significant differences in antibody level changes between patients who did and who did not develop severe infections (Table 6).

**Figure 4:**
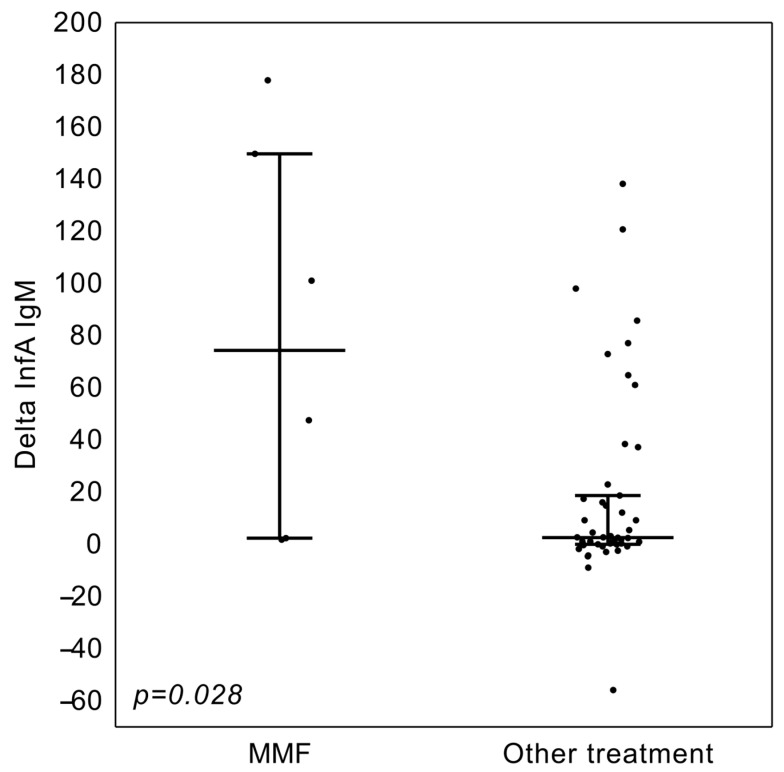
Change of Anti-Influenza virus A IgM class antibodies levels in the vKTx group in regard to MMF/MPA therapy status. The line denotes the median, whereas whiskers span the interquartile range (Q1-Q3).

We noticed that higher changes in antibody levels were observed in KTx who were not administered MMF/MPS only in the Anti-Influenza virus A IgM class (Table 7, Figure 4).

## 4. Discussion

Despite influenza being a highly contagious disease that kills nearly half a million people worldwide annually and despite the availability of well-known preventive vaccinations, the vaccination rate, including KTx recipients, remains still low [11]. Although influenza vaccination in our kidney transplant recipients did not entirely prevent influenza infection, vKTx patients developed a less severe course of the disease as compared to nvKTx patients. We also observed a tendency to the less use of antibiotics and significantly lower incidence of flu-like symptoms in vKTx patients’ family members, known as “cocooning strategy”, in comparison with non-vaccinated kidney recipients.

SOT recipients, in particular, are at increased risk of severe influenza, making vaccination a life-saving option for them. Nevertheless, the rates of vaccination in KTx recipients fall short of national goals, with influenza coverage reported at around 50% [12].

Our study indicates that the efforts of transplant healthcare providers can significantly increase seasonal vaccination rates among KTx recipients, as proven in our study group, where intensive promotion of vaccination made the vaccination rate go up as high as 72% in comparison to only 36% a year earlier. In the literature, recommendations from healthcare professionals are often cited as the primary reason for receiving vaccination. Thus, lack of or insufficient clinician advice can contribute to a decreased vaccination rate, along with such factors as anti-vaccine movement, patient hesitancy, or concern about side effects [15].

Many studies have found that influenza vaccination is safe post-transplant, both in terms of graft function and general side effects associated with the injection [16,17]. The most common adverse reactions to vaccination include pain and tenderness at the injection site, followed by systemic symptoms like headache, fatigue, general malaise, and muscle or joint pain, which were also observed in our patients, albeit less frequently than reported in the literature [18]. No serious adverse effects, such as urticaria, angioedema, or anaphylaxis, were noted in our cohort, although such rare reactions can occur after influenza vaccination, even among individuals with no previous reactions or known allergies [18]. Detailed analysis by The Vaccine Adverse Event Reporting System (VAERS) indicates that the occurrences of anaphylaxis and other allergic reactions may reflect an underlying predisposition to allergy that could manifest after exposure to any drug or vaccine and does not necessarily suggest that quadrivalent inactivated influenza vaccine is particularly allergenic [18].

There is limited data regarding the rejection or appearance of anti-HLA antibodies after influenza vaccination [7]. Some studies report increases in histological cellular rejection or detection of new donor-specific anti-HLA antibodies after vaccination, with or without clinical rejection or change in graft function [8]. However, other studies have shown no increased risk of allograft dysfunction including clinical rejection [7]. Conversely, not vaccinating may leave transplant recipients vulnerable to infection, potentially affecting graft and patient survival and leading to allograft dysfunction. Moghaddasi et al. observed no significant difference in serum creatinine, creatinine clearance, and 24-hour urine proteinuria levels between before and 1 month after influenza vaccination [17]. In our cohort, we noticed no de novo signs of graft injury, such as newly developed proteinuria or decreased eGFR after vaccination. The graft function in our vaccinated patients was stable, but we did not evaluate the donor-specific HLA antibodies either before or after vaccination.

While influenza vaccination has proven to be the most effective measure to reduce influenza virus infection in SOT recipients [4,10], these patients remain vulnerable due to their lower immunological response to vaccination compared to the general population [10]. Brakemeier et al. reported that vaccine responses in KTx recipients depend on the timing of vaccination relative to transplantation surgery, the type of immunosuppressive regimen, and graft function [8]. Our patients were at least 6 months after transplantation, and they received a standard immunosuppressive regimen with TAC trough level (C0 5–8 ng/mL) or CsA (C0 75–125 ng/mL) [19]. We did not observe any significant differences in response to influenza vaccination between patients who were treated with tacrolimus as compared to the cyclosporine A regimen.

While interpreting the results of the study, one must take into consideration that our assessment of infections was limited and based on the occurrence of symptoms instead of targeted antigen/PCR tests for influenza A/B infection.

Mycophenolate, due to its inhibition of lymphocyte B function, has been shown to be a particularly potent inhibitor of vaccine immunogenicity [20]. High doses of MMF/MPS inhibit influenza-induced CD86 and human leucocyte antigen-DR expression on B cells, resulting in a poor response to influenza vaccine [20]. Most of our patients were administered MMF/MPS, and the drug was not suspended for the time of vaccination. In six patients who were not treated with mycophenolate, we noticed a tendency for higher Anti- Influenza A virus antibody changes as compared to mycophenolate treatment.

Despite the lower serological response described in the literature, vaccinated KTx recipients present significantly reduced infection-related hospitalization and mortality compared to those who have not been vaccinated [21]. Moreover, healthcare workers and family members of transplant recipients should be fully immunized, including annual influenza vaccines. This strategy, known as “cocooning vaccination”, aims to protect vulnerable individuals who cannot be effectively vaccinated by creating a circle of immunity through the immunization of their close contacts. The benefits of cocooning vaccination extend beyond a lower risk of infection to include non-quantifiable factors such as an increased sense of security [21]. In our study, the “cocooning vaccination” syndrome was confirmed. The infectious symptoms among vKTx patients’ families and/or coworkers were reported significantly less often than those of non-vaccinated individuals.

The recommended vaccine for population vaccination is the quadrivalent inactivated influenza vaccine, which requires annual re-administration due to rapid antigenic drift, especially in influenza A (H3N2) viruses, necessitating reconfiguration of the vaccine [22]. Jones-Gray et al. concluded that the currently available data suggest vaccine effectiveness to be comparable for people vaccinated in both the current and previous season and for people vaccinated in the current season only, and better than for people vaccinated in the previous season only, indicating that vaccination in successive seasons offers better protection against influenza illness than no vaccination [23].

Thus, to improve influenza vaccine immunogenicity, especially in the vulnerable immunosuppressed SOT population, some researchers have reported a booster effect of the seasonal influenza vaccine [24].

Our patients were vaccinated with the most popular in Poland quadrivalent influenza vaccine, VAXIGRIP TETRA, providing active immunization against four influenza virus strains (two A subtypes and two B types). We administered a standard intramuscular influenza vaccine with no booster dose.

The Advisory Committee on Immunization Practices (ACIP) currently recommends only one dose of the standard quadrivalent inactivated influenza vaccine or quadrivalent recombinant influenza vaccine for SOT patients [22]. Several trials investigating booster doses, higher doses, or adjuvanted pandemic vaccines were evaluating their ability to overcome the relatively low immunogenicity of inactivated influenza vaccines in SOT. However, the best vaccination strategy and optimal timing for influenza vaccination in organ recipients remain unclear [4].

Not surprisingly, influenza vaccine immunogenicity is influenced by age, sex, concomitant diseases, immune status, and pre-existing influenza immunity. The most established correlate of protection against influenza virus infection is the presence of hemagglutination-inhibition (HAI) antibodies. The immunogenicity of a vaccine is typically based on seroconversion rates or the development of seroprotective titers [25]. Although in our study we did not assess HAI antibodies, which is one of the limitations of the study, we observed a statistically significant increase of Anti-Influenza A virus IgM antibodies as well as Anti-Influenza B virus IgG antibodies in the vKTx group at TP2 as compared with the measurement before vaccination. When assessed according to ELISA cut-off points for positive, inconclusive, or negative results for particular antibodies, the patient’s status remained comparable between TP1 and TP2. However, antibody serum concentration provided more conclusive results. Thus, the assessment of exact antibody concentration can be interpreted as favorable for protection from the development of severe infection, which consequently decreases the necessity of antibiotic use. It is of note that influenza vaccination is also crucial for patients with autoimmune diseases under immunosuppressive therapies. Murdaca et al. assessed that immunization against influenza is safe and immunogenic for immunocompromised patients with scleroderma or systemic lupus erythematosus, leading to a seroconversion rate comparable to healthy controls without presenting severe adverse reactions [26,27].

## 5. Conclusions

Influenza vaccination is an effective and safe procedure in recipients for a long period after kidney transplantation. It prevents the severe course of influenza and gives the “cocooning vaccination” effect. Despite immunosuppressive therapy, most patients present a serological response to influenza vaccination. Further studies should discuss the results and how they can be interpreted from the perspective of previous studies and of the working hypotheses. The findings and their implications should be discussed in the broadest context possible. Future research directions may also be highlighted.

## Figures and Tables

**Table 1:** Baseline vKTx and nvKTx group characteristics.

Variable	All (*n =* 110)	vKTx (*n =* 79)	nvKTx (*n =* 31)	*p*	No Data
Age [years]	54.91 (44.87–62.35)	55.31 (44.81–62.54)	53.58 (44.87–60.67)	0.98	-
Time from KTx [years]	5.98 (3.26–8.25)	5.75 (3.10–8.00)	6.77 (3.91–11.04)	0.12	-
Age at KTx [years]	47.36 (38.28–57.09)	46.29 (38.28–57.81)	47.53 (37.03–53.91)	0.68	-
Height [cm]	173.0 (165.0–178.0)	175.0 (167.0–178.5)	167.0 (162.0–175.0)	0.001	8
Weight [kg]	81.0 (70.0–90.0)1	83.5 (74.0–93.0)	70.5 (62.0–85.5)	0.002	12
BMI [kg/m^2^]	27.12 (23.80–30.10)	27.92 (24.51–30.45)	24.03 (22.11–28.55)	0.01	15
eGFR [mL/min/1.73 m^2^]	53.9 (39.9–69.3)	52.8 (35.1–64.3)	58.4 (49.1–71.3)	0.10	1
sCr [mg/dL]	1.39 (1.09–1.78)	1.52 (1.17–1.87)	1.19 (0.99–1.59)	0.01	1
Proteinuria > 0.5 g/L	12.15% (*n =* 13)	17.11% (*n =* 13)	0% (*n =* 0)	0.01	3

**Table 2:** Baseline vKTx and nvKTx group characteristics.

Variable	All Patients (*n =* 110)	vKTx (*n =* 79)	nvKTx (*n =* 31)	OR	95% CI	*p*	No Data
Sex (male)	78 (70.9%)	61 (77.2%)	17 (54.8%)	2.79	1.16–6.74	0.02	-
History of influenza vaccination	40 (36.4%)	29 (36.7%)	11 (35.5%)	1.05	0.44–2.51	0.90	-
Comorbidities							
Diabetes	33 (30%)	24 (30.4%)	9 (29.0%)	1.06	0.31–2.23	0.96	-
Ischemic heart disease	106 (96.4%)	76 (96.2%)	30 (96.8%)	0.84	0.09–8.44	0.88	-
Lung disease	7 (6.4%)	5 (6.3%)	2 (6.5%)	0.64	0.12–3.57	>0.99	3
History of heart infarction	8 (7.3%)	5 (6.3%)	3 (9.7%)	0.41	0.09–1.86	0.68	3
History of stroke	9 (8.2%)	7 (8.9%)	2 (6.5%)	0.92	0.18–4.81	>0.99	3
Immunosuppressive treatment							
Prednisone	108 (98.2%)	79 (100%)	29 (93.6%)	0	-	0.08	-
Tacrolimus	88 (80%)	65 (82.3%)	23 (74.2%)	1.62	0.6–4.35	0.36	-
Everolimus	6 (5.5%)	4 (5.1%)	2 (6.5%)	0.77	0.13–4.45	>0.99	-
Cyclosporine A	19 (17.3%)	14 (17.7%)	5 (16.1%)	1.12	0.37–3.43	>0.99	-
Mycophenolate mofetil/ mycophenolate sodium	93 (84.6%)	67 (84.8%)	26 (83.9%)	1.07	0.34–3.35	0.79	-

**Table 3:** Incidence of infections in vKTx and nvKTx groups.

Variable	vKTx (*n =* 79)	nvKTx (*n =* 31)	OR	95% CI	*p*
Mild infection	18 (22.8%)	6 (19.4%)	1.23	0.44–3.46	0.7
No. of mild infections per patient					
1	12 (15.2%)	3 (9.7%)			
2	4 (5.1%)	2 (6.5%)			
3	1 (1.3%)	1 (3.2%)	-	-	0.76
4	0	0			
5	1 (1.3%)	0			
Severe infection	9 (11%)	10 (32%)	0.27	0.10–0.75	0.01
No. of severe infections per patient					
1	7 (8.9%)	9 (29.0%)			
2	1 (1.3%)	1 (3.3%)	-	-	0.01
3	1 (1.3%)	0			
Any infection (mild or severe)	25 (32%)	14 (45%)	0.56	0.24–1.32	0.2
Total no. of infections	41	21			
Antibiotic therapy per patient	6 (7.6%)	6 (19%)	0.34	0.10–1.16	0.09
No. of antibiotic uses per patient with infection					
1	6 (25%)	4 (28.6%)			
2	0	1 (7.1%)	-	-	0.07
3	0	1 (7.1%)			
Antibiotic therapy per infection	8 (19.5%)	9 (42.9%)	0.32	0.10–1.03	0.06
Occurrence of similar infectious symptoms among coworkers or family members per infection	10 (24.4%)	12 (57.1%)	0.24	0.07–0.74	0.01
Hospitalization	3 (3.8%)	1 (3.2%)	1.18	0.12–11.84	0.99

**Table 4:** Multivariable logistic regression model with stepwise variable selection.

Variable	Reference Level	β	OR (95%CI)	*p*
Intercept		−1.281	0.28 (0.07–1.13)	0.07
Time after KTx [years]		0.140	1.15 (1.00–1.32)	0.04
Vaccination	Yes vs. No	−1.002	0.37 (0.19–0.70)	0.01
Sex	F vs. M	−0.577	0.56 (0.27–1.15)	0.11
Lung disease	Yes vs. No	1.097	2.99 (1.04–8.61)	0.04
Diabetes	Yes vs. No	0.673	1.96 (1.02–3.76)	0.04

**Table 5:** Anti-Influenza virus A and B antibody titers at TP1 and TP2.

Variable		Total	vKTX	nvKTx
TP1				
Anti-Influenza A virus IgM	Positive	28 (43.8%)	24 (46.2%)	4 (33.3%)
	Inconclusive	11 (17.2%)	9 (17.3%)	2 (16.7%)
	Negative	25 (39.1%)	19 (36.5%)	6 (50.0%)
Anti-Influenza A virus IgG	Positive	64 (100.0%)	52 (100.0%)	12 (100.0%)
	Inconclusive	0 (0%)	0 (0%)	0 (0%)
	Negative	0 (0%)	0 (0%)	0 (0%)
Anti-Influenza B virus IgM	Positive	9 (14.1%)	8 (15.4%)	1 (8.3%)
	Inconclusive	9 (14.1%)	7 (13.5%)	2 (16.7%)
	Negative	46 (71.9%)	37 (71.2%)	9 (75.0%)
Anti-Influenza B virus IgG	Positive	62 (96.9%)	51 (98.1%)	11 (91.7%)
	Inconclusive	0 (0%)	0 (0%)	0 (0%)
	Negative	2 (3.1%)	1 (1.9%)	1 (8.3%)
TP2				
Anti-Influenza A virus IgM	Positive	35 (54.7%)	31 (59.6%)	4 (33.3%)
	Inconclusive	12 (18.8%)	11 (21.6%)	1 (8.3%)
	Negative	17 (26.6%)	10 (19.2%)	7 (58.3%)
Anti-Influenza A virus IgG	Positive	64 (100.0%)	52 (100.0%)	12 (100.0%)
	Inconclusive	0 (0%)	0 (0%)	0 (0%)
	Negative	0 (0%)	0 (0%)	0 (0%)
Anti-Influenza B virus IgM	Positive	11 (17.2%)	9 (17.3%)	2 (16.7%)
	Inconclusive	7 (10.9%)	6 (11.5%)	1 (8.3%)
	Negative	46 (71.9%)	37 (71.6%)	9 (75.0%)
Anti-Influenza B virus IgG	Positive	62 (96.9%)	51 (98.1%)	11 (91.7%)
	Inconclusive	1 (1.6%)	0 (0.0%)	1 (8.3%)
	Negative	1 (1.6%)	1 (1.9%)	0 (0.0%)

**Table 6:** Changes in Anti-Influenza virus antibody levels depending on patient sex, comorbidities, and severe infection development.

Variable		Change in Antibody Level TP1 to TP2	*p*
Anti-Influenza A virus IgM			
Severe infection (*n =* 45)	Yes (*n =* 5)	9.19 (5.41–16.04)	0.40
	No (*n* = 40)	2.45 (0.12–42.95)	
Sex (*n =* 45)	M (*n =* 34)	2.14 (0.28–38.39)	0.39
	F (*n =* 11)	9.19 (2.34–47.51)	
Diabetes (*n =* 45)	Yes (*n =* 14)	6.16 (1.77–38.39)	0.40
	No (*n* =31)	2.41 (-0.05–61.02)	
Hypertension (*n =* 45)	Yes (*n =* 43)	2.63 (0.28–47.51)	0.80
	No (*n =* 2)	5.83 (2.48–9.19)	
Lung disease (*n =* 45)	Yes (*n =* 4)	7.30 (2.95–36.98)	0.62
	No (*n =* 41)	2.48 (0.28–38.39)	
Anti-Influenza A virus IgG			
Severe infection (*n =* 45)	Yes (*n =* 5)	13.5 (1.86–119.17)	0.79
	No (*n* =40)	22.48 (1.82–44.35)	
Sex (*n =* 45)	M (*n =* 34)	18.39 (1.86–45.63)	0.34
	F (*n =* 11)	34.46 (0.52–112.92)	
Diabetes (*n =* 45)	Yes (*n =* 14)	20.27 (-6.42–41.14)	0.33
	No (*n* =31)	22.58 (4.89–48.57)	
Hypertension (*n =* 45)	Yes (*n =* 43)	20.41 (0.52–45.63)	0.29
	No (*n =* 2)	88.13 (22.58–153.68)	
Lung disease (*n =* 45)	Yes (*n =* 4)	14.82 (3.54–25.53)	0.41
	No (*n =* 41)	22.58 (1.86–46.10)	
Anti-Influenza B virus IgM			
Severe infection (*n =* 45)	Yes (*n =* 5)	−0.10 (−0.38–2.95)	0.77
	No (*n* = 40)	0.05 (−0.36–0.86)	
Sex (*n =* 45)	M (*n =* 34)	0.19 (−0.14–1.10)	0.01
	F (*n =* 11)	−0.47 (−1.18–(−0.15))	
Diabetes (*n =* 45)	Yes (*n =* 14)	−0.22 (−1.18–0.66)	0.20
	No (*n* = 31)	0.17 (−0.22–1.10)	
Hypertension (*n =* 45)	Yes (*n =* 43)	−0.02 (−0.40–0.98)	0.29
	No (*n =* 2)	1.57 (0.19–2.95)	
Lung disease (*n =* 45)	Yes (*n =* 4)	−0.10 (−1.23–0.71)	0.53
	No (n = 41)	0.02 (−0.31–0.98)	
Anti-Influenza B virus IgG			
Severe infection (*n* = 45)	Yes (*n =* 5)	42.12 (−8.98–45.61)	0.96
	No (*n =* 40)	22.44 (2.98–42.40)	
Sex (*n* = 45)	M (*n =* 34)	20.72 (2.13–43.12)	0.19
	F (*n =* 11)	31.66 (9.22–56.34)	
Diabetes (*n =* 45)	Yes (*n =* 14)	28.45 (7.47–53.78)	0.28
	No (*n* =31)	19.01 (2.13–42.12)	
Hypertension (*n =* 45)	Yes (*n =* 43)	22.45 (2.22–45.61)	0.77
	No (*n =* 2)	26.17 (10.21–42.12)	
Lung disease (*n =* 45)	Yes (*n =* 4)	22.45 (2.34–42.12)	0.77
	No (*n =* 41)	26.17 (−1.87–94.26)	

**Table 7:** Changes in Anti-Influenza virus antibody levels depending on mycophenolic acid treatment.

Variable	Me (Q1–Q3)		*p*
	MMF (*n =* 6)	Other Treatment (*n* = 52)	
Delta Anti-Influenza A virus IgM	74.28 (2.34–149.68)	2.56 (0.00–18.64)	0.03
Delta Anti-Influenza A virus IgG	26.11 (20.41–48.57)	21.25 (0.52–45.63)	0.70
Delta Anti-Influenza B virus IgM	0.91 (−0.19–11.21)	0.00 (−0.47–1.10)	0.26
Delta Anti-Influenza B virus IgG	53.78 (25.23–83.25)	20.72 (2.13–42.12)	0.11

## Data Availability

Dataset available on request from the authors.

## Abbreviations

The following abbreviations are used in this manuscript:

SOT	solid organ transplant
KTx	kidney transplantation
vKTx	vaccinated kidney transplant recipient
nvKTx	non-vaccinated kidney transplant recipient
GFR	glomerular filtration rate
MDRD	modification of diet in renal disease
TP1	timepoint 1
TP2	timepoint 2
TP3	timepoint 3
AUC	area under the curve
MMF	mycophenolate mofetil
MPS	mycophenolate sodium
CsA	cyclosporine A
TAC	tacrolimus
mTOR	mammalian target of rapamycin
VAERS	The Vaccine Adverse Event Reporting System
ACIP	The Advisory Committee on Influenza
HAI	hemagglutination-inhibition
